# Network Pharmacology Analysis and Experiments Validation of the Inhibitory Effect of JianPi Fu Recipe on Colorectal Cancer LoVo Cells Metastasis and Growth

**DOI:** 10.1155/2020/4517483

**Published:** 2020-07-25

**Authors:** Xinyi Lu, Xingli Wu, Lin Jing, Lingjia Tao, Yingxuan Zhang, Renke Huang, Gong Zhang, Jianlin Ren

**Affiliations:** Department of Oncology, Shanghai Municipal Hospital of Traditional Chinese Medicine, Shanghai University of Traditional Chinese Medicine, Shanghai 200071, China

## Abstract

**Objective:**

To analyze the active compounds, potential targets, and diseases of JianPi Fu Recipe (JPFR) based on network pharmacology and bioinformatics and verify the potential biological function and mechanism of JPFR *in vitro* and *in vivo*.

**Methods:**

Network pharmacology databases including TCMSP, TCM-PTD, TCMID, and DrugBank were used to screen the active compounds and potential drug targets of JPFR. Cytoscape 3.7 software was applied to construct the interaction network between active compounds and potential targets. The DAVID online database analysis was performed to investigate the potential effective diseases and involved signaling pathways according to the results of the GO function and KEGG pathways enrichment analysis. To ensure standardization and maintain interbatch reliability of JPFR, High Performance Liquid Chromatography (HPLC) was used to establish a “chemical fingerprint.” For biological function validation, the effect of JPFR on the proliferation and migration of CRC cells *in vitro* was investigated by CCK-8 and transwell and wound healing assay, and the effect of JPFR on the growth and metastasis of CRC cells *in vivo* was detected by building a lung metastasis model in nude mice and *in vivo* imaging. For the potential mechanism validation, the expressions of MALAT1, PTBP-2, and *β*-catenin in CRC cells and transplanted CRC tumors were detected by real-time PCR, western blot, and immunohistochemical staining analysis.

**Results:**

According to the rules of oral bioavailability (OB) > 30% and drug-likeness (DL) > 0.18, 244 effective compounds in JPFR were screened out, as well as the corresponding 132 potential drug targets. By the analysis of DAVID database, all these key targets were associated closely with the cancer diseases such as prostate cancer, colorectal cancer, bladder cancer, small cell lung cancer, pancreatic cancer, and hepatocellular carcinoma. In addition, multiple signaling pathways were closely related to JPFR, including p53, Wnt, PI3K-Akt, IL-17, HIF-1, p38-MAPK, NF-*κ*B, PD-L1 expression and PD-1 checkpoint pathway, VEGF, JAK-STAT, and Hippo. The systematical analysis showed that various active compounds of JPFR were closely connected with Wnt/*β*-catenin, EGFR, HIF-1, TGF*β*/Smads, and IL6-STAT3 signaling pathway, including kaempferol, isorhamnetin, calycosin, quercetin, medicarpin, phaseol, spinasterol, hederagenin, beta-sitosterol, wighteone, luteolin, and isotrifoliol. For *in vitro* experiments, the migration and growth of human CRC cells were inhibited by the JPFR extract in a dose-dependent way, and the expression of MALAT1, PTBP-2, *β*-catenin, MMP7, c-Myc, and Cyclin D1 in CRC cells were downregulated by the JPFR extract in a dose-dependent way. For *in vivo* metastasis experiments, the numbers of lung metastasis were found to be decreased by the JPFR extract in a dose-dependent manner, and the expressions of metastasis-associated genes including MALAT1, PTBP-2, *β*-catenin, and MMP7 in the lung metastases were downregulated dose dependently by the JPFR extract. For the orthotopic transplanted tumor experiments, the JPFR extract could inhibit the growth of orthotopic transplanted tumors and downregulate the expression of c-Myc and Cyclin D1 in a dose-dependent manner. Moreover, the JPFR extract could prolong the survival time of tumor-bearing mice in a dose-dependent manner.

**Conclusions:**

Through effective network pharmacology analysis, we found that JPFR contains many effective compounds which may directly target cancer-associated signaling pathways. The *in vitro* and *in vivo* experiments further confirmed that JPFR could inhibit the growth and metastasis of CRC cells by regulating *β*-catenin signaling-associated genes or proteins.

## 1. Introduction

Traditional Chinese medicine (TCM) has become an important method for comprehensive treatment of different cancer patients. Clinical studies and meta-analyses indicated that longer duration of TCM herbal use is associated with improved survival outcomes, lower incidence of neurotoxicity, and higher quality of life in later period colorectal cancer patients in China [1–3]. JianPi Fu Recipe (JPFR) is a TCM compound from long-term clinical experience, which is composed of *Astragalus propinquus* Schischkin (Leguminosae), *Sophora flavescens* Aiton (Leguminosae), *Codonopsis pilosula* (Franch.) Nannf (Campanulaceae), *Atractylodes japonica* Koidz. ex Kitam. (Compositae), *Poria cocos* (Schw) Wolf (Polyporaceae), *Epimedium brevicornu* Maxim. (Berberidaceae), *Pinellia ternata* (Thunb.) Makino (Araceae), *Citrus reticulata* Blanco (Rutaceae), *Glycyrrhiza uralensis* Fisch (Leguminosae), and *Curcuma phaeocaulis* Valeton (Zingiberaceae).

Chinese herbal formulae are complicated in compounds, targets, and action mechanisms. The emergence of databases, such as TCMSP [4], TCMID [5], TCM-PTD [6], and DrugBank [7] and the development of network pharmacology [8, 9] and bioinformatics [10] provide new directions to screen the active components and potential targets and predict the function and mechanism of JPFR. Wu et al. applied the methods of molecular docking and computer network pharmacology to screen the active ingredients of Chinese herbs for coronary heart disease treatment and constructed a drug-target-disease network to investigate the regularity of TCM on the complex network in the body [11]. Li et al. applied the network pharmacology method to explain the material bases of the cold syndrome-heat syndrome [12–15]. This study firstly aims to explore the effective active compounds, effective targets, and involved pathways of JPFR basing on multiple online databases and network pharmacology analysis and verify the potential biological function and mechanism of JPFR *in vitro* and *in vivo*, providing scientific evidence for the clinical application of JPFR.

## 2. Materials and Methods

### 2.1. Network Pharmacology Analysis of JPFR

The active compounds of JPFR were mainly mined from the online databases TCMSP (http://lsp.nwsuaf.edu.cn/tcmsp.php), TCMID (http://www.megabionet.org/tcmid), and TCM-PTD (http://tcm.zju.edu.cn/ptd). All the targets of the screened active compounds were obtained from Drugbank 6.8 database (http://tcm.zju.edu.cn/ptd). Cytoscape software 3.7 was applied to construct the active compound-target network for JPFR. The KEGG enrichment analysis was performed using the DAVID database, including biological functions and signaling pathways. Top 12 cancers and 12 KEGG enrichment pathways were listed in graphs (*P* < 0.05). Based on the active compound-target network of JPFR constructed above and the searching of literatures, the systematical analysis of the potential regulatory signaling pathways of the most active compounds was provided.

### 2.2. Cell Line and Cell Culture

The colorectal cancer cell line used in this study was LoVo (human colon, Dukes' type C, grade IV, colorectal adenocarcinoma) from ATCC. The culture medium contained F-12K (SIGMA, UK) supplemented with 10% FBS and 100 U/ml penicillin and 100 g/ml streptomycin. LoVo cells were incubated in a couveuse at 37°C with 5% CO_2_, with high humidity.

### 2.3. Preparation of the JianPi Fu Recipe Extract

JianPi Fu Recipe (JPFR) is composed of dried medicinal herbs including *Astragalus propinquus* Schischkin (Leguminosae), *Sophora flavescens* Aiton (Leguminosae), *Codonopsis pilosula* (Franch.) Nannf (Campanulaceae), *Atractylodes japonica* Koidz. ex Kitam. (Compositae), *Poria cocos (*Schw) Wolf (Polyporaceae), *Epimedium brevicornu Maxim.* (Berberidaceae), *Pinellia ternata* (Thunb.) Makino (Araceae), *Citrus reticulata* Blanco (Rutaceae), *Glycyrrhiza uralensis* Fisch (Leguminosae), and *Curcuma phaeocaulis* Valeton (Zingiberaceae). The preparation of the alcohol extract of JPFR is divided into two steps: firstly, soaking dried traditional Chinese herbal medicine with 10 times volume 95% ethanol for 1 hour and, then, heating reflux extraction for 1 hour, filtrating them and, secondly, adding 8 times volume 95% ethanol reflux extraction for 1 hour, repeating the aforementioned procedure. Finally, these two filtered solutions are combined, ethanol is retrieved, and we get the JPFR alcohol extract. To prepare 20 mg/ml JPFR solution, a 0.24 g JPFR alcohol extract was weighed using electronic scales and dissolved in a 12 ml F-12K medium supplemented with 10% fetal bovine serum (FBS), following by ultrasonic mixing solution overnight and filtering bacteria with a 0.22 *μ*m filter. In the following experiments, we diluted the 20 mg/ml JPFR solution to the required concentration using the F-12K medium supplemented with 10% FBS.

### 2.4. High Performance Liquid Chromatography (HPLC) Analysis

HPLC analysis was conducted by using the Agilent 1260 infinity (ZK-001).The separation was carried out on an Alltech Uitrasphere C18 column. In order to establish the High Performance Liquid Chromatography (HPLC) method for the determination of astragaloside IV, matrine, and oxymatrine in JPFR, an Alltech Uitrasphere C18 (Alltech Ultrasphere C18) was used with acetonitrile-water (32 : 68) as the mobile phase. The flow rate was 1.0 ml/min, and the detection wavelength was at 220 mm. The column temperature was 30°C.

JPFR was handled by low temperature drying and, then, made into lyophilized powder. The precisely weighed 5 g lyophilized powder was added into 20 ml purified water. It was carefully weighed again after adding water, followed by ultrasonic processing for 30 minutes. Then, it is weighed again, and purified water is used to make up to the lost weight. We waited until the powder was fully dissolved. The solution was filtered, and the 25 ml overfiltrate was precisely measured. The saturated n-butanol was used to extract the overfiltrate 4 times, 25 ml each time. The n-butanol solution was combined and washed 3 times with ammonia solution, 30 ml each time, and the washing solution was discarded. The n-butanol solution was evaporated, the residue was dissolved with methanol, transferred to a 5 ml volumetric flask, methanol was added to scale, and shook well. All the tested solutions were filtered through Millex 0.22 *μ*m nylon membrane syringe filters (Millipore, USA). The contents of astragaloside IV, matrine, and oxymatrine in each sample were calculated using standard curves of astragaloside IV, matrine, and oxymatrine.

### 2.5. CCK-8 Analysis

The LoVo cells in the logarithmic growth period were inoculated in 96-well plates at the concentration of 2000–3000 cells per well. After the cells completely adhered, the serum-free medium was changed to starve overnight. Adding the alcohol extract of JPFR, the concentration of JPFR was prepared with 12.5 *μ*g/mL, 25 *μ*g/mL, 50 *μ*g/mL, 100 *μ*g/mL, 200 *μ*g/mL, 300 *μ*g/mL, 400 *μ*g/mL, and 500 *μ*g/mL. A control group was established. After 48 hours, the cellcountingkit-8 solution was added to each pore at the ratio of 10%. After the solution mixed completely, cells were cultured at 37°C for 4 hours. The absorption of the enzyme was measured at 490 nm/630 nm (double wavelength).

### 2.6. Colony Formation Analysis

The cells which were starved for 2 days were incubated with JPFR in a conditioned medium for 6 days. After washing in the conditioned medium 2 times, the cells were suspended in the conditioned medium. The cell suspension concentration was adjusted to 1 × 10^3^/ml. The cell suspension of 0.4 ml per well was cultured in a 6-well culture plate for 2 weeks, and the colony formation was observed. When the cells formed visible clones (about 50–150 cells per clone), cells were terminated cultured. The cells were incubated with PBS 2 times, and then, carbinol 1 ml/well was added. After fixing for 15 min, 1ml giemsa was added to each hole for 30 mins. The culture plate was placed in the gel imaging system. With the visible light condition, the number of clones was counted by software carried by the machine. Images were scanned and saved. Clone formation rate = clone number/number of inoculated cells × 100%. Adjusted clone formation rate = Clone formation rate (%)/Clone formation rate in the control group (%).

### 2.7. Transwell Assay

The serum-free medium was diluted with 1 : 4, and 40 *μ*L was added to the room of the 24-orifice plate transwell chamber, and the room temperature was maintained overnight until the gel solidified. The surface of the transwell chamber was incubated on the surface of the membrane with fibronectin as a cytochemotactic factor. 100 *μ*L, 1 × 10^5^ cell suspension that resuspended in a serum-free medium was added to the transwell chamber, and the next chamber was added the medium of 10% fetal bovine serum. The cells in each group had 3 complex holes, and the methods of observation and analysis were the same as those of the migration experiment.

### 2.8. Wound Healing Assay

Cells (4 × 10^5^) in the 10% FBS-containing medium were seeded on a six-well plate to make a confluent monolayer. The monolayer cells were scratched by a plastic tip and washed with PBS to remove cell debris. An F-12K medium containing 0.5% FBS was, then, added to each well, and the scratched monolayer was incubated in a 37°C incubator with 5% CO_2_ for 24 hours. Wound closure was measured in five random fields at ×200 magnification using a DMI3000B inverted microscope (Leica, USA). The wound healing percentage was calculated as follows: migrated cell surface area/total surface area × 100%, in which migrated cell surface area = length of cell migration (mm) × 2 × length of defined areas, total surface area = beginning width × length of defined areas.

### 2.9. *In Vivo* Experiment

For the lung metastasis model, the single-cell suspension was prepared by the logarithmic long-term fluorescent LoVo cells, and the cell concentration was diluted to 2.5 × 10^7^/ml by PBS (phosphate buffer saline). After the activity of cells was evaluated more than 95% by trypan blue dyeing, 200 *μ*L cell suspension was injected to the tail vein of BALB/c nude mice. The growth and metastasis of tumor in the body were observed by using the American fine real living imaging system (IVIS 200). Each nude mouse was injected with 150 *μ*L 30 mg/ml fluorescein substrate 10–25 min before observation.

For the orthotopic transplantation tumor model, the single-cell suspension was prepared by the LoVo cells from the logarithmic growth period, and the cell concentration was adjusted to 1 × 10^7^/ml by PBS. After the activity of cells was evaluated more than 95% by trypan blue dyeing, the orthotopic transplantation tumor model began to establish. 10 BALB/c nude mice (4-6-week old, 20 g ± 2 g body weight), half male and half female, were prepared. The subcutaneous of the armpit of the right forelimb of nude mice was routinely sterilized. After modeling, the size of tumor in the tumor-bearing mice was observed, and the tumor grew to about 1–1.2 cm in diameter. After being executed, the tumor was removed and cut into 1 mm^3^ and, then, inoculated in the cecum of nude mice. After the operation, the abdominal junction was sutured with an operative thread. Under the condition of SPF (Specific Pathogen Free) in an isolated squirrel cage, it was kept at constant temperature and humidity.

Forty mice were prepared for the lung metastasis model and orthotopic transplantation tumor model, respectively. For each model, the mice were randomly divided into 5 groups, 8 mice in each group: (1) a blank control group (model group): intragastric administration of normal saline (once a day, for 7 days); (2) a low-dose JPFR group: JPFR decoction gavage (once a day, a total of 28 days); (3) a medium dose JPFR group: JPFR decoction gavage (once a day, a total of 28 days); (4) a high-dose JPFR group: JPFR decoction gavage (once a day, a total of 28 days); and (5) a positive control group (L-OHP): intraperitoneal injection of oxaliplatin 0.6 mg/kg mL (once every other day, for 28 days). After 28 days, the nude mice were sacrificed. The primary tumors and the lung tissues of the mice were surgically removed and investigated. All experimental protocols were reviewed and approved by the animal ethics committee of Shanghai Municipal Hospital of Traditional Chinese Medicine, Shanghai University of Traditional Chinese Medicine.

### 2.10. Real-Time PCR

The total RNA of cells was extracted by the one-step method by Trizol, and then, 2 *μ*g was used for reverse transcription. Quantitative PCR was carried out using the 20 *μ*l reaction system. The sample tube and the internal tube were equipped with a double tube. The gene expression levels were analyzed by quantitative real-time PCR using the ABI7500 instrument (ABI, USA). The reaction conditions were 94°C for 2 min, 94°C for 30 s, 60°C for 30 s, and a total of 40 cycles. After the end of the reaction, ABI 7500 SDS software automatically analyzed the fluorescence signal and converted it to the Ct value. The sequences of the primers used are listed in Table 1.

### 2.11. Western Blot

Cells were washed by PBS three times, and then, the cell lysate was added. The extracted proteins were quantified and loaded for SDS-PAGE gel electrophoresis, transferred to PVDF membranes, and blocked with 10% milk. Subsequently, the membranes were incubated with the primary antibodies and the following HRP-conjugated secondary antibodies. All the resulting immunocomplexes were visualized by enhanced chemiluminescence, followed by directly photographing and quantitative analyzing.

### 2.12. Immunohistochemical Staining Analysis

Paraffin-embedded tissues were sectioned for IHC (immunohistochemistry) staining. In detail, the experiments were performed using the first antibody, HRP-conjugated secondary antibody, and DAB (diaminobenzidine) detection reagents. The DMI3000B microscope was used for photographing. All the data were evaluated and classified blindly by two investigators from the pathology department of Shanghai Municipal Hospital of Traditional Chinese Medicine, Shanghai University of Traditional Chinese Medicine.

### 2.13. Statistical Analysis

The data were represented by means ± SD or median with 95% CI (confidence interval). Statistic analysis was performed using Student's *t*-test, one-way AVOVA analysis, the Mann–Whitney test, or the Kruskal-Wallis test, as appropriate, with the significance level at *P* < 0.05. The results were analyzed by SPSS 22.0 software (IBM, USA).

## 3. Results

### 3.1. Potential Effect Mechanism Mining of JPFR Basing on Network Pharmacology Analysis

**Figure 1:**
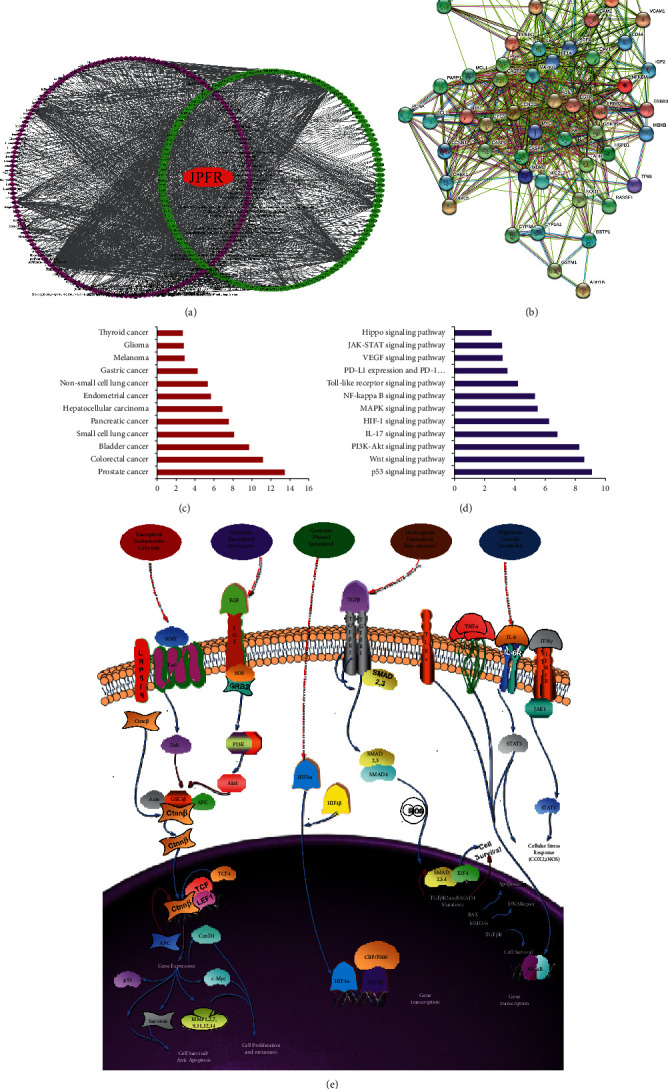
Potential effect mechanism mining of JPFR basing on network pharmacology analysis. (a) Active compounds of JPFR were screened using the network pharmacology databases, including TCMSP, TCMID, and TCM-PTD. TCMSP and Drugbank database were used to screen the targets for each active compound in JPFR. The compound-target network was constructed by Cytoscape 3.7 software. The red octagon represents the traditional Chinese medicine compound, the purple circle represents the compound, the green diamond represents the target, and the black line represents the relationship between the compound active ingredients and the corresponding targets. (b) PPI (Protein-Protein Interaction) Network Diagram according to screening results from (a). The circles in the PPI network represent the interaction of different targets for JPFR, and the lines represent interactions with each other. (c-d) DAVID database was used to mine the effective cancer diseases and potential signaling pathways of JPFR. Fifty-five target genes were enriched in the GO function and KEGG signaling pathway analysis. (e) Systematical analysis of the potential regulatory mechanism of active ingredients basing on the active compound-target network of JPFR constructed before and the searched literatures.

Active compounds of JPFR were screened using the network pharmacology databases, including TCMSP, TCMID, and TCM-PTD. The results showed that a total of 245 active ingredients were screened out in JPFR according to the cutoff value of OB > 30% and DL > 0.18, including 20 compounds in *Astragalus propinquus* Schischkin (Leguminosae), 7 compounds in *Atractylodes japonica* Koidz. ex Kitam. (Compositae), 5 compounds in *Citrus reticulata* Blanco (Rutaceae), 21 compounds in *Codonopsis pilosula* (Franch.) Nannf. (Campanulaceae), 92 compounds in *Glycyrrhiza uralensis* Fisch (Leguminosae), 13 compounds in *Pinellia ternata* (Thunb.) Makino (Araceae), 15 compounds in *Poria cocos* (Schw.) Wolf (Polyporaceae), 45 compounds in *Sophora flavescens* Aiton (Leguminosae), 23 compounds in *Epimedium brevicornu Maxim.* (Berberidaceae), and 3 compounds in *Curcuma phaeocaulis* Valeton (Zingiberaceae) (Table S1). TCMSP and Drugbank database were used to screen the targets for each active compound in JPFR. We have found 953 targets in *Astragalus propinquus* Schischkin (Leguminosae), 774 targets in *Atractylodes japonica* Koidz. ex Kitam. (Compositae), 479 targets in *Citrus reticulata* Blanco (Rutaceae), 911 targets in *Codonopsis pilosula* (Franch.) Nannf. (Campanulaceae), 2506 targets in *Glycyrrhiza uralensis* Fisch (Leguminosae), 1302 targets in *Pinellia ternata* (Thunb.) Makino (Araceae), 121 targets in *Poria cocos* (Schw.) Wolf (Polyporaceae), 963 targets in *Sophora flavescens* Aiton (Leguminosae), 24 targets in *Curcuma phaeocaulis* Valeton (Zingiberaceae), and 510 targets in *Epimedium brevicornu* Maxim. (Berberidaceae). By removing duplicate targets, 132 targets were screened for the following analysis (Table S1). To determine the relationship between the compounds in 10 Chinese herbal medicines of JPFR and the corresponding targets, a compound-target network was constructed by Cytoscape 3.7 software.  Figure 1(a) shows that the compound-target network includes 10 herbs, 244 compounds, and 132 targets in total. In the network, some targets interact with more compounds than others. This suggests that many targets can be regulated by a variety of compounds rather than just one compound. For example, prostaglandin G/H synthase 1/2 (PTGS1/2), peroxisome proliferator-activated receptor gamma (PPARG), serine/threonine-protein kinase Chk1 (CHEK1), and glycogen synthase kinase-3*β* (GSK-3*β*) are composed of various active substances.

As shown in Figure 1(b), the circles in the PPI network represent the interaction of different targets for JPFR, and the lines represent interactions with each other. The network consists of 54 nodes and 532 edges, indicating that there are 532 relationships among the 54 target genes. The more the number of connected lines (i.e., the degree), the more important the targets in the network, such as the targets CASP3 (degree = 41), VEGFA (degree = 41), EGFR (degree = 40), IL6 (degree = 40), and MAPK8 (degree = ) with a large median value (not less than 30). 40) and CCND1 (degree = 39), MYC (degree = 39), ERBB2 (degree = 33), and CYCS (degree = 39).

DAVID database was used to mine the potentially effective diseases of JPFR. Fifty-four target genes were enriched in the GO function and KEGG pathway enrichment analysis. According to the statistical principle *P* < 0.05, the KEGG enrichment analysis results show that the top 12 cancer diseases that JPFR might have potential effective efficacy are presented in Figure 1(c), such as prostate cancer, colorectal cancer, bladder cancer, small cell lung cancer, pancreatic cancer, and hepatocellular carcinoma. The DAVID Functional Annotation database was applied to further explore the molecular mechanism of the active compounds of JPFR in different cancer diseases. The data showed that multiple signaling pathways including p53, Wnt, PI3K-Akt, IL-17, HIF-1, p38-MAPK, NF-*κ*B, PD-L1 expression and PD-1 checkpoint pathway, VEGF, JAK-STAT, and Hippo were closely related to JPFR (Figure 1(d)). Based on the active compound-target network of JPFR constructed before, the systematical analysis showed that various active compounds of JPFR, such as kaempferol, isorhamnetin, calycosin, quercetin, medicarpin, phaseol, spinasterol, hederagenin, beta-sitosterol, wighteone, luteolin, and isotrifoliol, were closely connected with Wnt/*β*-catenin, EGFR, HIF-1, TGF*β*/Smads, and IL6-STAT3 signaling pathway (Figure 1(e)).

### 3.2. Effects of JPFR on the Proliferation and Migration of CRC Cells *In Vitro*

**Figure 2:**
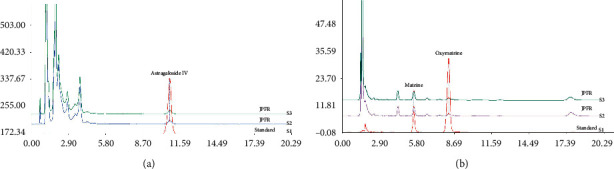
Quality control of JPFR extracts basing on the HPLC analysis of astragaloside IV, matrine, and oxymatrine. HPLC (High Performance Liquid Chromatography) analysis was performed to characterize the ingredients of astragaloside IV in Astragalus propinquus Schischkin (Leguminosae)(one component of JPFR) and matrine and oxymatrine in Sophora flavescens Aiton (Leguminosae)(one component of JPFR).

**Figure 3:**
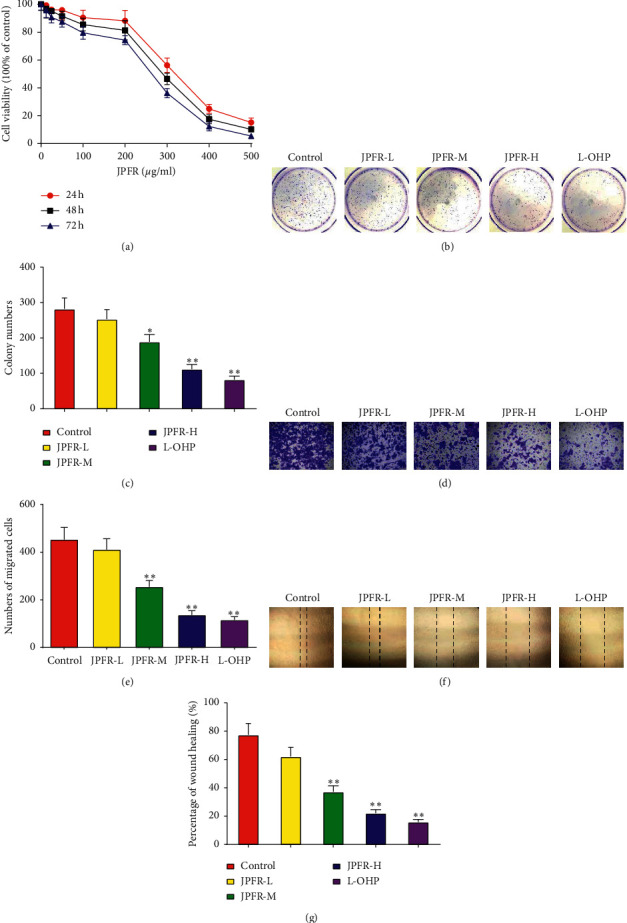
Effects of JPFR on the proliferation and migration of CRC cells *in vitro*. (a) CCK-8 assay was performed to observe the effect of JPFR on the proliferation of CRC cells. (b, c) Colony formation and quantitative analysis were performed to observe the effect of JPFR on the colony-forming ability of CRC cells. (d, e) Transwell and quantitative assay were performed to evaluate the migration ability of LoVo cells. (f, g) Wound healing and quantitative assay were performed to evaluate the migration ability of LoVo cells. ^*∗*^*P* < 0.05, ^*∗∗*^*P* < 0.01 versus the control group.

Our clinical practice has demonstrated that JPFR can reduce distant metastasis of CRC and improve the life quality of CRC patients. Therefore, herein, we focus our research on the underlying function and mechanism of JPFR in CRC cells. As shown in Figure 2, HPLC data indicated that JPFR has a stable ingredient of astragaloside IV, matrine, and oxymatrine, indicating the consistent quality of JPFR for the following *in vitro* and *in vivo* experiments. Firstly, the CCK-8 assay was performed to observe the effect of JPFR on the proliferation of CRC cells. The results showed that JPFR has an obvious inhibitory effect on the proliferation of LoVo cells in a dose-dependent manner (Figure 3(a)). The subsequent colony formation experiments further confirmed that JPFR can inhibit the colony-forming ability of CRC cells in a dose-dependent manner (Figures 3(b) and 3(c)).

Subsequently, the transwell and wound healing experiments were performed to see the effect of JPFR on the migration of CRC cells. The transwell assay demonstrated that JPFR inhibited the migration of LoVo cells in a concentration-dependent manner (Figures 3(d) and 3(e)). In addition, the wound healing assay further demonstrated that JPFR could inhibit the migration of LoVo cells in a concentration-dependent manner (Figures 3(f) and 3(g)). All the abovementioned data demonstrated that JPFR has obvious inhibitory effects on the proliferation and migration of CRC cells *in vitro*.

### 3.3. Effects of JPFR on CRC Metastasis- and Growth-Related Genes and Proteins *In Vitro*

**Figure 4:**
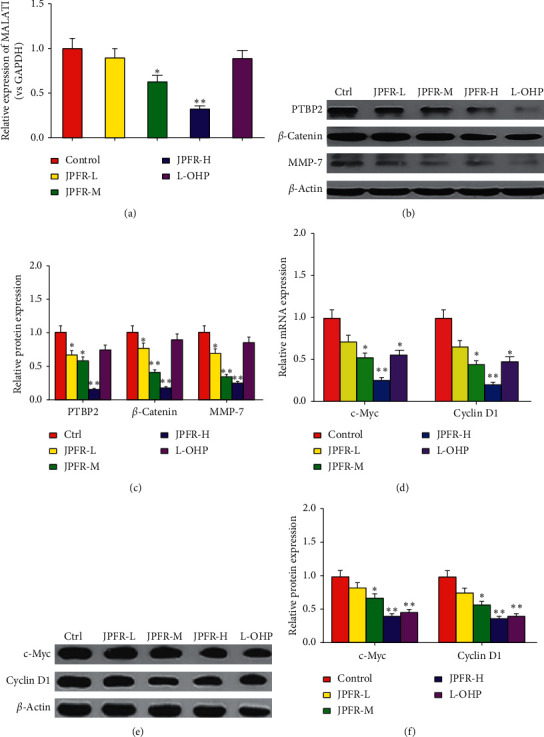
Effects of JPFR on CRC metastasis- and growth-related genes and proteins *in vitro*. (a) Real-time PCR was performed to observe the effect of JPFR on the relative MALAT1 expression. (b-c) Western blot was performed to study the effect of JPFR on the protein expression of PTBP-2, *β*-catenin, and MMP7. (d) Real-time PCR was performed to observe the effect of JPFR on the relative mRNA expression of c-Myc and Cyclin D1. (e-f) Western blot was performed to study the effect of JPFR on the protein expression of c-Myc and Cyclin D1. ^*∗*^*P* < 0.05, ^*∗∗*^*P* < 0.01 versus the control group.

Since JPFR has inhibitory effects on the proliferation and migration of CRC cells *in vitro*, we further investigated whether JPFR could affect the expression of some metastasis- and growth-related genes and proteins. In Table 2, by network pharmacology analysis, we have showed the intersection genes between JPFR and intestinal cancer targets, such as GSK3B, CCND1, HIF-1A, MYC, CCNB1, and CD44, implying the vital regulatory effect of JPFR on Wnt/*β*-catenin signaling. MALAT1 (metastasis-associated lung adenocarcinoma transcript 1) is a lncRNA that plays an important role in the growth and metastasis of CRC and associated closely with Wnt/*β*-catenin signaling [16]. Quantitative real-time PCR was used to observe the effects of JPFR on the expression levels of MALAT1 in CRC cells. The results showed that JPFR could decrease the mRNA levels of MALAT1 in a dose-dependent manner (Figure 4(a)). PTBP-2, *β*-catenin, and MMP7 have been reported as the downstream targeted proteins of MALAT1 [16, 17]. Our western blot assay further demonstrated that JPFR could downregulate the expression of PTBP-2, *β*-catenin, and MMP7 in a dose-dependent manner (Figures 4(b) and 4(c)).

c-Myc and Cyclin D1 are both important regulators for tumor growth [18, 19]. Our quantitative real-time PCR data demonstrated that JPFR could decrease the mRNA expression of c-Myc and Cyclin D1 in a dose-dependent manner (Figure 4(d)). Western blot assay further demonstrated that JPFR could downregulate the protein expression of c-Myc and Cyclin D1 in a dose-dependent manner (Figures 4(e) and 4(f)). All the abovementioned results demonstrated that JPFR inhibited the proliferation and migration of CRC cells partly through regulating the expression of MALAT1 and downstream targets PTBP-2, *β*-catenin, and MMP7, as well as c-Myc and Cyclin D1.

### 3.4. Effects of JPFR on CRC Lung Metastasis in Nude Mice

**Figure 5:**
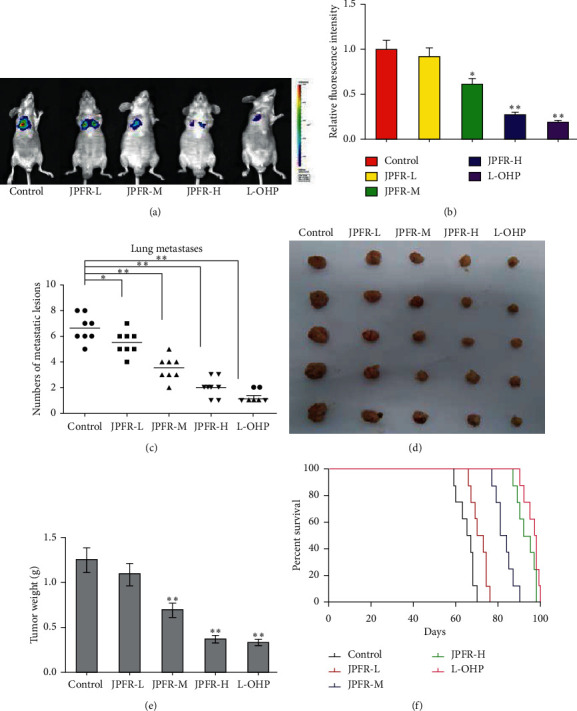
Effects of JPFR on CRC lung metastasis and orthotopic transplanted tumors growth in nude mice. (a-c) Effect of JPFR on the lung metastasis of human colon cancer cells in nude mice. (d-e) Effect of JPFR on the orthotopic transplanted CRC tumors growth in nude mice. ^*∗*^*P* < 0.05, ^*∗∗*^*P* < 0.01 versus the control group. (f) Effect of JPFR on the survival time of nude mice with orthotopic transplanted CRC tumors.

In order to investigate the inhibitory effects of JPFR on lung metastases *in vivo*, the experimental lung metastasis model was built in nude mice by tail vein injection of Luc-labeled CRC LoVo cells. In Figures 5(a) and 5(b), the luciferase intensity of Luc-labeled LoVo cells in lung metastatic lesions in the positive control L-OHP group was significantly decreased relative to the blank control group. The treatment of low-dose JPFR can decrease the luciferase intensity of Luc-labeled LoVo cells in lung metastatic lesions. Moreover, the luciferase intensity of Luc-labeled LoVo cells in lung metastatic lesions in middle-dose and high-dose JPFR groups were found to be dramatically decreased, compared to the control group and low-dose JPFR group. After 28 days, the nude mice were sacrificed, and the lung metastatic lesions of the mice were surgically removed and investigated. The results in Figure 5(c) demonstrated that JPFR reduced the numbers of lung metastatic lesions in a dose-dependent manner relative to the control group. In addition, the positive control L-OHP group also significantly reduced the numbers of lung metastatic lesions relative to the blank control group (Figure 5(c)).

### 3.5. Effects of JPFR on the Orthotopic Transplanted Tumors Growth of CRC in Nude Mice

To observe the effects of JPFR on the tumors growth of CRC *in vivo*, we built the orthotopic transplanted tumors model by implanting the subcutaneous tumor into the cecum of the nude mice. After administration of JPFR for 7 weeks, parts of the nude mice were killed and the tumors were weighed. In Figures 5(d) and 5(e), the results showed that JPFR could significantly reduce the sizes and weights of orthotopic transplanted tumors of CRC in a dose-dependent manner. In addition, our results showed that JPFR could significantly increase the survival time of nude mice with orthotopic transplanted tumors of CRC in a dose-dependent manner (Figure 5(f)). All the abovementioned data implied that JPFR can inhibit the growth of orthotopic transplanted tumors of CRC and prolong the survival time of tumor-bearing mice.

### 3.6. Regulatory Effects of JPFR on Metastasis- and Growth-Related Genes and Proteins *In Vivo*

**Figure 6:**
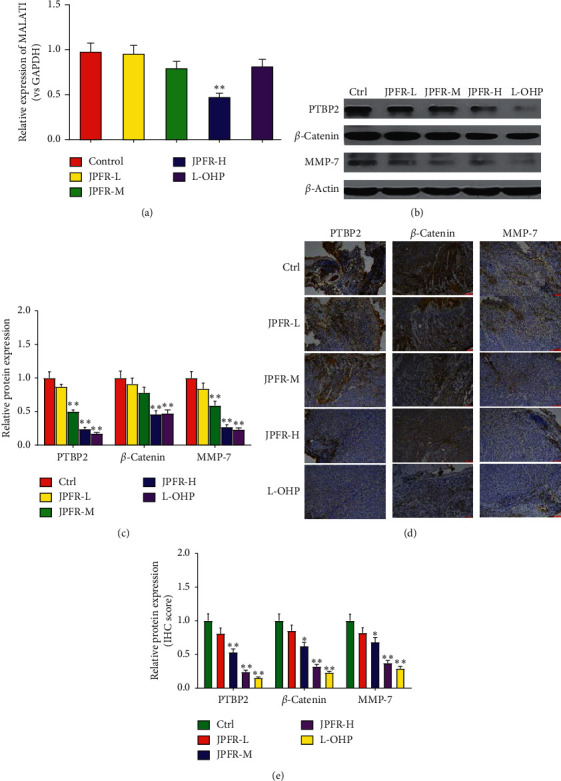
Regulatory effects of JPFR on metastasis-related genes and proteins in vivo. (a) Real-time PCR assay of the expression level of MALAT1. (b-c) Western blot and quantitative analysis of the protein expressions of PTBP2, *β*-catenin, and MMP7 in the lung metastases. (d-e) Immunohistochemistry and quantitative analysis of the expressions of PTBP-2, *β*-catenin, and MMP7 in the lung metastases. ^*∗*^*P* < 0.05, ^*∗∗*^*P* < 0.01 versus the control group.

Quantitative real-time PCR was used to detect the lncRNA expression of the metastasis-related gene MALAT1 in the lung metastases. The results showed that JPFR could decrease the lncRNA expression of MALAT1 in a dose-dependent manner (Figure 6(a)). Western blot assay further demonstrated that JPFR could downregulate the expression of MALAT1 downstream targeted proteins PTBP-2, *β*-catenin, and MMP7 in a dose-dependent manner (Figures 6(b) and 6(c)). This result was further confirmed by the immunohistochemical staining detection of PTBP-2, *β*-catenin, and MMP7 in the lung metastases (Figures 6(d) and 6(e)).

**Figure 7:**
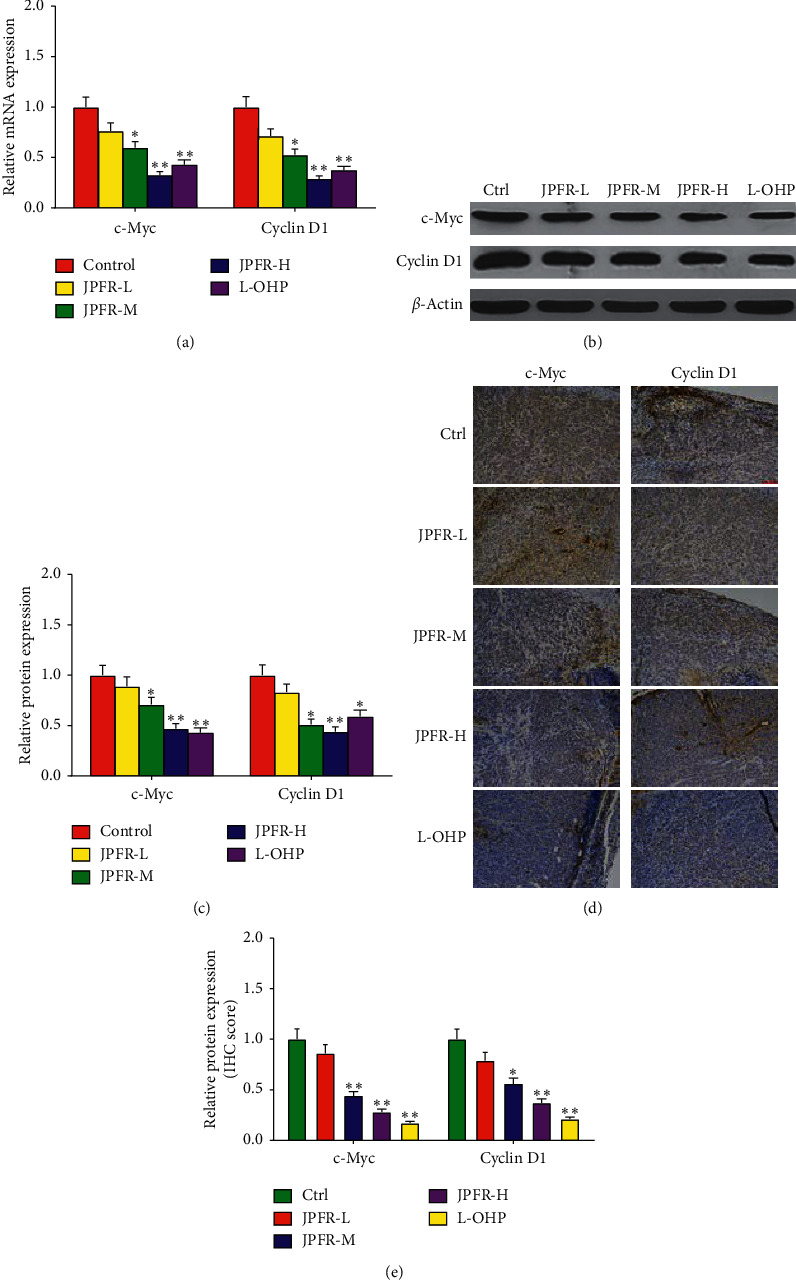
Regulatory effects of JPFR on tumor growth-related genes and proteins *in vivo*. (a) Real-time PCR assay of the expression level of c-Myc and Cyclin D1. (b-c) Western blot and quantitative analysis of the protein expressions of c-Myc and Cyclin D1 in the orthotopic transplanted tumors. (d-e) Immunohistochemistry and quantitative analysis of the protein expressions of c-Myc and Cyclin D1 in the orthotopic transplanted tumors. ^*∗*^*P* < 0.05, ^*∗∗*^*P* < 0.01 versus the control group.

In addition, the quantitative real-time PCR results showed that JPFR could dose-dependently decrease the mRNA expression of c-Myc and CyclinD1 in the orthotopic transplanted tumors (Figure 7(a)). Western blot assay further demonstrated that JPFR could downregulate the expression of c-Myc and CyclinD1 in a dose-dependent manner (Figures 7(b) and 7(c)), which was further confirmed by immunohistochemical staining detection of c-Myc and CyclinD1 in the orthotopic transplanted tumors (Figures 7(d) and 7(e)).

## 4. Discussion

In the past few years, traditional Chinese medicine (TCM) has become an important method for comprehensive treatment of cancer patients. JianPi Fu Recipe (JPFR) is a TCM compound from long-term clinical experience, which is composed of *Astragalus propinquus* Schischkin (Leguminosae), *Sophora flavescens* Aiton (Leguminosae), *Codonopsis pilosula* (Franch.) Nannf (Campanulaceae), *Atractylodes japonica* Koidz. ex Kitam. (Compositae), *Poria cocos* (Schw) Wolf (Polyporaceae), *Epimedium brevicornu* Maxim. (Berberidaceae), *Pinellia ternata* (Thunb.) Makino (Araceae), *Citrus reticulata* Blanco (Rutaceae), *Glycyrrhiza uralensis* Fisch (Leguminosae), and *Curcuma phaeocaulis* Valeton (Zingiberaceae). The clinical application of TCM in cancer diseases needs the material basis support, which promotes better understanding of the underlying effect mechanism of JPFR in CRC treatment.

Generally, TCM is complicated in compounds, targets, and molecular mechanism. The emergences of databases including TCMSP [4], TCMID [5], TCM-PTD [6], and DrugBank [7], as well as the development of network pharmacology [8, 9] and bioinformatics [10], provide new strategies to screen the active components and potential targets and predict the potential mechanism of JPFR. In our study, we firstly explore the effective active compounds, effective targets, and involved pathways of JPFR based on multiple online databases and network pharmacology analysis. According to the rules of oral bioavailability (OB) > 30% and drug-likeness (DL) > 0.18, 244 effective compounds in JPFR were screened out, as well as the corresponding 132 potential drug targets. By the analysis of DAVID database [20], all these key targets were associated closely with the cancers such as prostate cancer, colorectal cancer, bladder cancer, small cell lung cancer, pancreatic cancer, and hepatocellular carcinoma. In addition, multiple signaling pathways were closely related to JPFR, including p53, Wnt, PI3K-Akt, IL-17, HIF-1, p38-MAPK, NF-*κ*B, PD-L1 expression and PD-1 checkpoint pathway, VEGF, JAK-STAT, and Hippo. The systematical analysis showed that various active compounds of JPFR were closely connected with Wnt/*β*-catenin, EGFR, HIF-1, TGF*β*/Smads, and the IL6-STAT3 signaling pathway, such as kaempferol, isorhamnetin, calycosin, quercetin, medicarpin, phaseol, spinasterol, hederagenin, beta-sitosterol, wighteone, luteolin, and isotrifoliol.

CRC is a heterogeneous and molecularly complex disease [21]. One of the leading challenges for CRC is that the clinical diagnosis of CRC may be late [22]. Approximately 20% of CRC patients have distant metastases at the time of diagnosis, and an additional 20% develop metastases during the follow-up [23]. The 5-year survival rate of distant metastases in CRC was only 12.5% [24]. Researchers strived to investigate the mechanisms to reduce the mortality of CRC in the past decades. Long noncoding RNAs (lncRNAs) are a group of noncoding RNAs composed of >200 nucleotides [25]. As an important lncRNA, metastasis-associated lung adenocarcinoma transcript 1 (MALAT1) has been demonstrated to be crucial in the processing of pre-mRNAs and was revealed to be upregulated in CRC [26]. It was found that MALAT1 could promote CRC growth and metastasis through regulating the MALAT1/PTBP-2/*β*-catenin signaling pathway. PTBP2 has been found highly expressed in cancer cells and can promote cancer cell proliferation [27, 28]. Galbán et al. found that PTBP2 and HUR jointly promoted the translation of hypoxia-inducible factor 1 *α* (HIF-1*α*) and regulated several downstream cancer-related genes, such as vascular endothelial growth factor (VEGF) and *β*-catenin [29]. Many studies have demonstrated that Wnt/*β*-catenin signaling connected closely with the progression and development of different cancers. Upon stimulation of upstream activators and the helping of some transcription factors such as BCL9, *β*-catenin protein translocates to the nucleus from the cytoplasm, binds to the promoter of TCF/LEF, and activates the transcription of its target genes such as c-Myc, cyclinD1, and MMP7, leading to tumor initiation and development [18, 19, 30, 31].

Clinical studies and meta-analyses have indicated that longer duration of TCM herbal use is associated with improved survival outcomes, lower incidence of neurotoxicity and higher quality of life in later period CRC patients in China [1–3]. Our clinical practice has demonstrated that JPFR can reduce distant metastasis of CRC and improve the life quality of CRC patients. For *in vitro* experiments, the migration and growth of human CRC cells were inhibited by JPFR extract in a dose-dependent way, and the expression of MALAT1, PTBP-2, *β*-catenin, MMP7, c-Myc, and Cyclin D1 in CRC cells was downregulated by JPFR extract in a dose-dependent way. For *in vivo* metastasis experiments, the numbers of lung metastasis were found to be decreased by JPFR extract in a dose-dependent manner, and the expressions of metastasis-associated genes including MALAT1, PTBP-2, *β*-catenin, and MMP7 in the lung metastasis were downregulated dose dependently by JPFR extract. For the orthotopic transplanted tumors experiments, JPFR extract could inhibit the growth of orthotopic transplanted tumors in a dose-dependent manner, and JPFR extract could downregulate the expression of c-Myc and CyclinD1 in a dose-dependent manner. Moreover, JPFR extract could prolong the survival time of tumor-bearing mice in a dose-dependent manner.

## 5. Conclusions

In summary, we demonstrated that JPFR contains many effective compounds which may directly target to the cancer-associated signaling pathways. The *in vitro* and *in vivo* experiments further confirmed that JPFR could inhibit the growth and metastasis of colorectal cancer LoVo cells by regulating *β*-catenin signaling-associated genes or proteins.

## Figures and Tables

**Table 1:** Primer sequences for real-time PCR.

Gene	Primer sequences	Amplified fragments (nt)
MALAT1	F:5-AGGCGTTGTGCGTAGAGGA-3 R:5-GGATTTTTACCAACCACTCGC-3	80
GAPDH	F:5-AGAAGGCTGGGGCTCATTTG-3 R:5-AGGGGCCATCCACAGTCTTC-3	258

**Table 2:** Intersection genes between JPFR and intestinal cancer targets.

Symbol	entrezID
PGR	5241
PTGS1	5742
AR	367
CHEK1	1111
CHRM3	1131
ADH1B	125
PPARG	5468
GSK3B	2932
IKBKB	3551
BCL2	596
CASP3	836
MAPK8	5599
CYP3A4	1576
CYP1A1	1543
ICAM1	3383
SELE	6401
VCAM1	7412
ALOX5	240
GSTP1	2950
AHR	196
GSTM1	2944
EGFR	1956
VEGFA	7422
CCND1	595
FOS	2353
CASP9	842
PLAU	5328
RB1	5925
IL6	3569
TP63	8626
NFKBIA	4792
CASP8	841
HIF1A	3091
ERBB2	2064
CAV1	857
MYC	4609
BIRC5	332
DUOX2	50506
NOS3	4846
HSPB1	3315
CCNB1	891
NFE2L2	4780
NQO1	1728
PARP1	142
CRP	1401
RASSF1	11186
IGF2	3481
ERBB3	2065
TIMP1	7076
MDM2	4193
PCNA	5111
MCL1	4170
CYCS	54205
CD44	960

## Data Availability

The data used to support the findings of this study are included within the article.

## Supplementary Materials

Supplementary Materials Table S1 is “potential effect mechanism mining of JPFR basing on network pharmacology analysis.” Other explanatory text of figures and tables has already been listed in the separated Word.
